# Prevalence and predictors of burnout among Indian healthcare professionals: A cross-sectional analytical study

**DOI:** 10.6026/973206300221622

**Published:** 2026-03-31

**Authors:** Varsha Raja Ayyanar, Prajin Kishore Pushparaj Virgin Rani

**Affiliations:** 1 Department of Urology, Royal Cornwall Hospital NHS Trust, Cornwall, Truro TR1 3LJ, United Kingdom

**Keywords:** Burnout, healthcare professionals, emotional exhaustion, organisational factors, maslach burnout inventory (MBI)

## Abstract

Burnout among healthcare workers is an escalating occupational health concern that adversely affects both provider well-being and patient care outcomes. Therefore, it is of interest to assess the prevalence and predictors of burnout among healthcare professionals using the Maslach Burnout Inventory (MBI). Of 400 recruited participants, 350 completed the assessment, with nurses demonstrating higher levels of emotional exhaustion and depersonalization compared to other professionals. Organizational factors such as flexible scheduling and access to mental health resources were associated with reduced burnout, while heavy workload and inadequate staffing contributed significantly. Thus, we show the need for targeted organizational interventions to mitigate burnout and improve healthcare workforce sustainability.

## Background:

Occupational burnout occurs in response to chronic stress related to work performance. Symptoms of occupational burnout are emotional exhaustion, depersonalization and a feeling of reduced personal accomplishment [1]. Healthcare workers are very much at risk for developing occupational burnout since they face a high level of emotional and physical demands from taking care of patients [2]. Examples of factors contributing to workplace stress are long shifts, high volume of patients and exposure to patients with life-threatening illnesses. The most current research indicates that global burnout rates have reached an alarming rate [3]. Burnout rates among healthcare workers are highest among nurses and doctors. The COVID-19 pandemic made the problem of occupational burnout worse by increasing the number of hours, uncertainty and emotional distress healthcare workers experienced [4]. Many healthcare professionals worked long hours, day after day, when they were exposed to a high level of risk and were often required to work short-staffed or no-staffed situations. Both healthcare worker and organizational outcomes are compromised by occupational burnout [5]. For example, healthcare professionals who experience burnout tend to have lower overall job satisfaction, are more likely to call in sick, are more likely to leave a job, are injured on the job and more likely are associated with patients who have negative outcomes from their experience while being treated in a hospital [6]. The seriousness of the negative effects of occupational burnout indicators on healthcare workers and organization makes it both an occupational health and a quality-of-care issue within the healthcare system [7]. The development of burnout is generally influenced by the organizational environment. Organizational work environments where employees work excessive hours, do not have adequate staff to share workload and are not supported by their organizations lead to burnout [8]. Therefore, it is of interest to assess the degree of incidences and predictors of burnout within the scope of practice of healthcare professionals in any district general hospital.

## Materials and Methods:

A cross-sectional analytical design was used in the present study to determine the geographic distribution of the prevalence of burnout in healthcare professionals employed by the District General Hospital. This study utilized mixed-methods to allow both quantitative and qualitative data to be collected. Thus, in this present study, data were gathered through a stratified, random sampling approach, which was based on a power analysis to determine an adequate number of respondents (*i.e.*, 400) to obtain adequate power; however, when accounting for non-responding participants, 350 completed surveys were analysed. Quantitative data were collected using a structured, self-administered survey, with an emphasis on using the Maslach Burnout Inventory (MBI) to assess emotional exhaustion, depersonalisation and personal achievement; in addition, participants were asked several questions about demographic information, department and strategies to reduce burnout in their workplace. The surveys used a 5-point Likert scale (1 = strongly disagree; 5 = strongly agree) to assess the level of burnout measured by participants, as well as their perception of the effectiveness of both individual and organisational support to reduce burnout. The target population for this study included doctors, nurses, allied health professionals and administrative staff working at the District General Hospital from various departments (*e.g.*, Emergency, ICU and Outpatient Services). To prospective participants, recruitment of the target population was conducted using a stratified random sampling technique. As previously mentioned, due to the use of a power analysis, a target of 400 respondents was established to ensure that enough respondents were recruited; however, once all non-responses were eliminated, there were 350 valid responses available for analysis.

## Results:

This research included 400 health care professionals from a variety of professions, including doctors, nurses, allied health professionals and administrators, who worked across all types of health care organizations (emergency, ICU, outpatient, *etc.*) and represented a variety of deliveries of health care services. The representatives of the four workforce categories were equally distributed: 45% of the respondents identified as nursing, 30% as physicians, 15% as allied health professionals and 10% as administrators. Overall, 75% of the respondents reported being in their current roles for less than ten years, represent both early-career and mid-career individuals. The study assessed burnout levels using the Maslach Burnout Inventory (MBI) to measure emotional exhaustion, depersonalization and perceived personal accomplishment. The nurse group had significantly higher average scores for emotional exhaustion (28.9±5.8) and depersonalization (14.3±5.0) than did the administrator group; whereas the administrator group had the lowest average score for emotional exhaustion (7.5±4.9). This suggests that administrators have lower levels of burnout than nurses. The evaluation of the accessibility of organizational strategies for addressing emotional exhaustion assessed strategies such as flexible scheduling, wellness programs, mental health resources and peer support. The strategy with the highest rating was flexible scheduling (4.2±0.9), followed closely by mental health resources (4.0±1.0). Inferential statistical analysis indicated that there was a statistically significant difference between job roles on emotional exhaustion scores (ANOVA F = 7.65, p < 0.001), suggesting that job role does influence on the scores of emotional exhaustions. The results of multiple regression analysis indicated that flexible scheduling and mental health resources were statistically significant predictors of lower emotional exhaustion (p < 0.05). Three main themes of the qualitative analysis of the data using thematic coding were identified: Staff workload/staffing; the need for supportive structures; autonomy/flexibility. Several staff members identified high patient loads along with insufficient staffing as being among the leading contributing factors to their experience of burnout. In addition, it was reported that the availability of a structured support program/mentorship as well as the opportunity to self-schedule work shifts are major factors in reducing stress. Through both qualitative and quantitative results, it was discovered that although organisational interventions have been shown to increase the perception of support, workload/staffing continue to be two significant contributing factors to burnout. These findings suggest that burnout results from a variety of systemic and individual factors and that targeted intervention techniques must be applied to remedy the relationship of workload issues; mental-health support; and workforce management to decrease burnout and improve the quality of patient-care in health-care facilities.

Table 1 (see PDF) provides information on the distribution of job roles among the respondents participated in the study. A total of 400 participants from the healthcare sector participated and the largest percentage of participants are nurses (45%), followed by doctors (30%), allied health professionals (15%) and administrative personnel (10%). Such as large number of nurses represents a significant part of the study and allows the study to reflect the perspective of numerous health care roles, with an emphasis on the direct clinical staff that are likely to be most affected by job burnout. Table 2 (see PDF) lists the distribution of participants' hospital departments represented in this study, with the largest group represented in outpatient (there were 30% of participants), Emergency (there were 25% of participants), other departments (there were 25% of participants) and ICU (there were 20% of participants). This disparity allows for the assessment of burnout in both high-stress environments (ICU and Emergency) as well as less acute environments, to determine how the respective workload and environment on departmental burnout influence on levels of burnout. Table 3 (see PDF) presents the participants' years of experience in their current role. Most respondents reported having from 0-10 years of work experience (75%). 30% of them were in the 0-5 year of experience range and 45% of them were in the 6-10 year of experience range, whereas 15% of the respondents stated having >11 years of work experience. This suggests that the sample for this study is composed mostly of early- to mid-career professionals, who may be experiencing different types of stress than other more experienced ones, including difficulties in adjusting to their workload and overall Organizational demands. Table 4 (see PDF) compares the average scores for emotional exhaustion, depersonalization and personal accomplishments based upon the employee's position. Staff in nursing averaged the highest of all roles for both emotional exhaustion (28.9±5.8) and depersonalization (14.3±5.0) scores which indicates an increased likelihood to experience burnout when compared to other employee job types. Conversely, Administrative staff averaged the lowest scores in emotional exhaustion and depersonalization indicating an overall lower level of emotional strain related to their jobs versus nursing staff. Administrative staff also averaged the highest in personal accomplishment (34.0±5.3) while nursing staff averaged the lowest (27.9±4.7) suggesting that nurses may have more difficulty in feeling accomplished than the higher-level administrative staff. In Table 5 (see PDF), the participants rated the effectiveness of a variety of interventions for addressing burnout. The mean rating for the effectiveness of flexible scheduling was the highest of any of the interventions (4.2±0.9) followed closely by that of mental health resources (4.0±1.0). Therefore, it appears that having choices and access to support for mental health help are seen as being important to the nursing staff. Wellness programs, work-life balance training and peer support were rated as somewhat effective in combating burnout. Therefore, these areas also represent an opportunity for improvement in the types of support provided by an employer to their staff. Additionally, Table 6 (see PDF) provides the regression analysis which identifies which factors specifically predict emotional exhaustion. Flexible scheduling (β = -0.31, p = 0.002) and mental health resources (β = -0.28, p = 0.004) were found to significantly decrease the amount of emotional exhaustion reported, thus, reinforcing the importance of having organizational supports to prevent burnout in healthcare employees. Job position/type (in particular being a nurse) was a significant predictor of burnout (β = 0.25, p = 0.01), thus, this supports the idea that nursing personnel have a higher probability of experiencing burnout than non-nursing personnel. Finally, while years of experience was not a significant predictor (β = 0.05, p = 0.42) of emotional exhaustion, this indicates that factors other than years of experience are more likely to contribute to an employee's likelihood of experiencing emotional exhaustion. Specifically, the level of job demand for nurses versus other healthcare workers combined with the level of organizational support available is more predictive than the number of years worked.

## Discussion:

The research indicated healthcare providers in various roles within a general community hospital experienced burnout; however, they did not know how to use the results to determine if their profession was affected more than other occupations. Based on the data collected, healthcare workers generally experienced more emotional and physical weariness than other professionals [9]. The study determined that nursing staff experienced significantly more emotional exhaustion and depersonalization than did other professionals, which was confirmed by previous research demonstrating that clinical staff, especially those on the front line, experienced the highest level of occupational stress [10]. The study also revealed that nurses continuously care for their patient's bedside, which allows them to build strong relationships with their patients, therefore creating additional emotional demands on their workdays, resulting in increased fatigue and emotional stress [11]. Conversely, administrative staff generally experienced lower levels of burnout compared to other professions because of less direct patient care and lower levels of physical and emotional stress [12]. The study results indicate that the role an employee plays within a healthcare organization greatly impacts their level of burnout and that every organization must look at the role of each employee when determining how to reduce burnout among their employees. In addition, the research established the tools provided by the organizations can help reduce their employees' emotional exhaustion [13]. Flexible scheduling and the availability of mental health services were significant predictors of employees with lower burnout levels. The findings are consistent with previous systematic reviews that provide evidence supporting the use of organizational interventions to address burnout [14]. Flexible scheduling creates a better work and family balance, so employees are less likely to experience fatigue. Mental health services provide emotional support, coping resources and timely intervention. Additionally, the qualitative findings supported the quantitative research results. Participants indicated that the primary cause of their burnout was a heavy workload, as well as a lack of sufficient staffing. These findings are consistent with the findings reported in global literature [15]. When there are too many patients for each staff member to care for, it creates stress and the recovery time for each staff member between consecutive workdays becomes inadequate. Continued workload pressure leads to an employee's emotional exhaustion and the employee's ability to build a relationship with their patients decreases or disappears [16]. Participants also indicated that having a supportive environment was critical for reducing their levels of burnout. Both mentoring programs and peer support from other healthcare providers were seen as very valuable. Participants also believed having autonomy and flexibility, to some extent, were both contributors to reducing their levels of burnout. Therefore, in order to reduce employee burnout and improve patient care, hospital systems require an organisational approach as well as a cultural change [17]. Several studies have revealed that burnout among healthcare providers has a significantly negative impact on the provision of quality, safe patient care. Previous research studies have also indicated a relationship between the occurrence of burnout and medical errors and patient satisfaction. Therefore, it is critical to address the issue of burnout among healthcare providers to improve health care quality and patient outcomes [18]. The results of this study indicate that for healthcare organisations to effectively address the issue of burnout, systemic interventions are required. Relying solely on providing resilience training to individual employees will not effectively address the problems associated with burnout. To adequately address the issues of workload, staffing and adequate levels of employee support systems, healthcare organisations must implement an organisational approach in addressing the issue of burnout [19]. The results of this study provide new evidence from a district general hospital, which together with the previous evidence contained in the literature, confirm that addressing healthcare provider burnout requires addressing multiple factors and that healthcare organisations must implement specific workplace interventions to effectively address burnout [20]. When evaluating this study, several limitations should be taken into account. As the study used a cross-sectional design, the findings of this study cannot be inferred for causal inferences regarding the prevalence of burnout among healthcare workers. In addition, the use of self-reported data may result in bias due to participant responses. However, the large sample size of healthcare workers lends strength to the results of the study. In conclusion, the results of this study clearly indicate the immediate need for healthcare organisations to implement effective organisational strategies to reduce the incidence of burnout among their employees. Supporting the healthcare workforce is a necessary component for both long-term sustainability of the workforce and safety for patients.

## Conclusion:

Healthcare workers, especially nurses, experience high rates of burnout due in large part to organizational factors. Workload demands, limited staffing levels and limited organizational support are major contributors to both emotional exhaustion and depersonalization. Flexible scheduling and access to mental health services through organizations are important protective factors. To address burnout, it is critical to implement systemic reform of the workplace instead of relying solely on individual strategies for coping.

## References

[1] Nguyen N (2025). Worldviews Evid Based Nurs..

[2] Maluleka MM (2026). S Afr Fam Pract..

[3] Puigtió-Rebollo N (2025). Nurs Open..

[4] Hostiuc S, Gherghiceanu F (2026). Medicina (Kaunas)..

[5] Maheshwari A (2025). J Assoc Physicians India..

[6] Daneshvar E, Otterbach S (2025). Sci Rep..

[7] Kapadia UH (2025). BMC Med Educ..

[8] Bolatov AK (2025). Sci Rep..

[9] Lee YL (2025). Sci Rep..

[10] Lee SY (2025). BMJ Open..

[11] Martinez-Calderon J, García-Muñoz C (2025). Curr Opin Oncol..

[12] Collett G (2025). J Occup Environ Med..

[13] Bondjers K (2025). BMC Health Serv Res..

[14] Amaya-Arias AC (2025). Biomedica..

[15] Muza M (2025). Med Sci Monit..

[16] Fournier A (2025). Acta Psychol (Amst)..

[17] Kmail ZM (2026). Am J Health Promot..

[18] Khan BM (2026). BMJ Open..

[19] Ahmed S (2026). BMC Med Educ..

[20] Ji W (2025). BMJ Open..

